# Maternal age and maternal environment affect stress reactivity and measures of social behaviour in laying hens

**DOI:** 10.1038/s41598-021-96323-6

**Published:** 2021-09-01

**Authors:** Mariana R. L. V. Peixoto, Leanne Cooley, Tina M. Widowski

**Affiliations:** 1 Department of Animal Biosciences, University of Guelph, Guelph, ON N1G 2W1 Canada; 2 L.H. Gray & Son Limited, Strathroy, ON Canada

**Keywords:** Animal behaviour, Animal physiology

## Abstract

Maternal effects can shape the phenotypes of offspring, but the extent to which a layer breeder’s experience can affect commercial laying hens remains unclear. We aimed to investigate the effects of maternal age and maternal environment on laying hens’ behaviour and stress response. In our first experiment (E1), commercial hybrid hens were reared either in aviary or barren brooding cages, then housed in aviary, conventional cages or furnished (enriched) cages, thus forming different maternal housing treatments. Hens from each treatment were inseminated at three ages, and measures of response to manual restraint and social stress were assessed in offspring. In experiment 2 (E2), maternal age effects on offsprings' stress response were further investigated using fertile eggs from commercial breeder flocks at three ages. In E1, maternal age affected struggling and corticosterone during manual restraint, feather pecking and pulling and comb wounds. Additionally, maternal rearing and housing in aviary systems showed positive effects on measures of behaviour and stress response in offspring. Effects of maternal age were not replicated in E2, possibly due to methodological differences or higher tolerance to maternal effects in commercial breeders. Overall, we recommend researchers report parent stock age to increase comparison across studies and thus our understanding of maternal age effects.

## Introduction

In birds, maternal experience can affect offspring phenotype through epigenetic changes that can occur during gametogenesis^1–3^ and by changes in egg composition during ovogenesis^4–6^. Within the latter, yolk hormones, specifically steroids, have been suggested to have a strong link with maternal effects, with both epigenetic and hormone-mediated effects observed on a vast array of physiological and behavioural traits^4^. In commercial egg production, the mothers of laying hens are derived from maternal lines of a single genetic strain and housed with males from the same strain’s paternal lines to produce the fertile eggs that are hatched and reared to establish the flocks of commercial laying hens. In their lifetimes, these layer breeders can produce approximately 115 female offspring^7^, meaning that their experiences can shape the phenotypes of a significant number of commercial laying hens.

Previous research has shown that hens subjected to a moderate heat challenge for five consecutive weeks laid eggs with higher concentrations of yolk steroid hormones (progesterone, testosterone and estradiol) and had lighter and calmer offspring than control^8^. In the Japanese quail, human behaviour during handling (predictable and gentle *versus* unpredictable and fast) also affected the concentration of progesterone and estradiol in yolk and on offspring social discrimination skills^9^. Likewise, Japanese quails subjected to stressful events, such as sudden movement or unpredictable noise, laid eggs with higher yolk testosterone and progesterone, and produced chicks more sensitive to social separation (i.e., vocalized more during emergence and open-field tests)^10^. Interestingly, stressful events applied onto five genetic lines of layer breeders decreased offspring’s sensitivity to social isolation in only one line, suggesting that maternal effects are also dependent on genetics^11^.

In the face of the global transition from conventional cages to cage-free systems, it is important to investigate the direct and indirect outcomes (e.g., maternal effects) of different housing systems for parent stock on the development, behaviour, and welfare of laying hens. Layer breeder parent stock are traditionally reared in small, barren conventional cages until 16–18 weeks of age (woa) and then transferred to either large cage-free groups on litter or smaller groups in colony cages^12^. However, due to concerns for animal welfare, we predict that the rearing of breeder flocks in aviaries will start to be discussed in the near future. Although there is a paucity of literature on the subject, a recent multifactorial analysis of laying rate, production of hatching eggs, and number of waste eggs showed that breeders reared in an aviary and subsequently transferred to litter had better results compared to cage-reared breeders^13^. From studies in commercial hens, it is known that the more spacious and complex aviary rearing improves the use of space^14^ and reduces fearfulness^15^, stress response^16^ and the number of eggs laid on litter^17^ in comparison to rearing in small, barren conventional brooding cages. Acute stress experienced during early life (around and before puberty) has also been shown to affect behaviour and stress response of offspring^18^. Adult housing also affects behaviour and stress response, with hens reared in conventional cages and housed in furnished cages producing less corticosterone at 50 woa and showing more comfort behaviour (preening) than hens both reared and raised in conventional systems^19,20^. It is, thus, clear that the environment can cause short- and long-term effects not only on production and physical traits but also on the behaviour and stress response of laying hens. Although a growing number of studies demonstrate effects of discrete acute stressors applied to laying hens on offspring behaviour and physiology, there have been no previous studies that have explored the more general effects of maternal housing environment during rearing and/or during the laying period on offspring traits. Therefore, investigating the link between maternal housing and offspring’s phenotype is a promising field of study that deserves attention.

Another emerging topic is the effect of maternal age on offspring behaviour. It is common knowledge among farmers that each flock is behaviourally unique, even when they come from the same genetic strain and provided identical nutrition and housing. One possible explanation is related to the mother’s age. Layer breeder flocks typically begin producing offspring at around 20 woa and remain in production until around 70 woa, reaching an optimized performance at approximately 44 woa^12,21^. As a hen gets older, the size of eggs increase and the quantity of solid content in her eggs naturally change^22^. Similarly, yolk testosterone concentration has been found to decrease with age both in layers^23^ and quails^24,25^, possibly affecting their offspring’s phenotype. Although the literature on the subject is still growing, previous research has shown that older quails (37 versus 11 woa) had offspring that showed less prominent emotional response to encountering a novel object and vocalized more in response to social isolation^24^. Interestingly, Cooley et al.^26^ found opposite results in layers, with a decrease in the vocalization pattern of chicks from hens at 68 woa compared to chicks of mothers who were 25 and 44 woa. Although further studies investigating possible pathways for these effects are still needed, the link between maternal age and offspring behaviour is unquestionably exciting and offers a new area to be explored.

The current study—which is part of a more comprehensive research project on maternal effects in laying hens^11,23,26–29^—investigates how maternal age, maternal rearing experience and maternal adult housing system affect their offsprings' behaviour and stress response. For this, we conducted two experiments. In the first (E1), we used commercial hybrid laying hens as a model to explore whether there are effects of maternal rearing environment, maternal adult housing environment or maternal age on their offspring. Two cohorts of Lohmann Selected Leghorn-Lite hens were reared and housed in five housing system combinations and were inseminated at three ages (25, 44 and 68 woa), producing six offspring flocks. Measurements of stress reactivity and injurious behaviour were assessed. In addition, since layer breeders seem to be more resilient to maternal effects than commercial layers^11,28^, and to test the replicability of the maternal age effects seen in E1, we conducted a second experiment (E2) using layer breeders of the same strain of hens, in which hatching eggs from three age groups [“Young” (25–27 woa), “Ideal” (42–46 woa) and “Old” (68–72 woa)] were obtained from various commercial flocks across Canada and the United States. Stress response was measured in offspring at 4 woa. We hypothesized that maternal effects related to age and environment would be found in E1 and predicted the replicability of maternal age effects in E2.

## Methods

This study was carried out in compliance with the ARRIVE guidelines and birds were treated in accordance with the Canadian Council on Animal Care. All procedures were approved by the University of Guelph Animal Care Committee (Animal Utilization Protocol #1947).

### Experiment 1 (E1)

#### Parent stock housing systems

Laying hens are reared and housed in a variety of artificial environments. These range from barren wire conventional cages, which provide little space, a relatively small group size and no environmental enrichment, to cage-free complex aviary systems where pullets and hens are kept in groups of hundreds to thousands, with nests, perches, and foraging substrate located on different levels. An intermediate housing system for laying hens is the furnished cage, in which the birds are provided more space than a conventional cage, a group size of 15 to 100, and environmental enrichments that include nests, low perches and foraging mats. Details of the rearing and adult housing used in E1 are provided in Supplementary Materials.

#### Parent stock management

In this experiment, 2 consecutive cohorts of 1356 Lohmann Selected Leghorn Lite (LSL-Lite) pullets each, were obtained from a commercial hatchery at one day of age and transferred to the University of Guelph’s Arkell Poultry Research Station. Birds within each cohort had been produced by a single breeding flock and were incubated, hatched and transferred to the research under identical conditions. Immediately upon arrival, part of the pullets from each flock were housed in conventional brooding cages (CC, n = 408), while the remaining was assigned to a tiered pullet aviary (Av, n = 778) system. At 16 weeks of age, pullets from Av rearing were transferred to conventional cages (CC; n = 6 cages of 8 hens), furnished cages (FC; n = 12 cages of 60 hens) and aviary systems (Av; n = 1 aviary groups of 370 hens). Pullets from CC rearing were transferred to CC (n = 12 cages of 8 hens) or FC (n = 12 cages of 60 hens).

**Figure 1:**
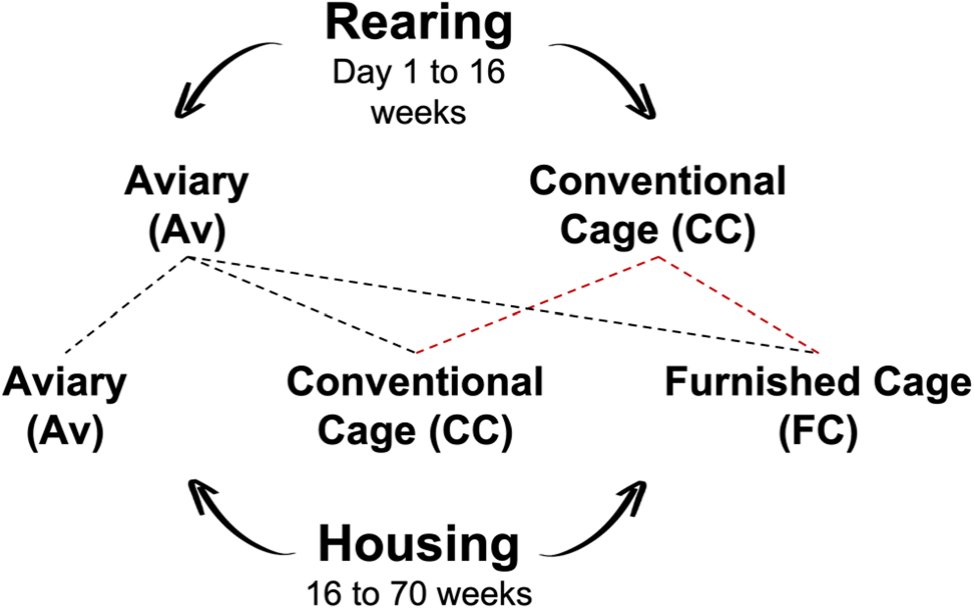
Experiment 1. The combination of maternal rearing and housing experiences formed five treatment groups: Trt1 (Av × Av), Trt2 (CC × CC), Trt3 (Av × CC), Trt4 (CC × FC), Trt5 (Av × FC). This was repeated for two cohorts of hens. Since pullets reared in cages lack experience with navigation in a 3-dimensional space^17^, only pullets reared in aviaries were transferred to aviary housing.

Each rearing and housing combination was considered a treatment (Fig. 1). A sample of 96 hens from each treatment were randomly selected and inseminated with pooled semen from a contemporary group of White Leghorn males at 3 ages: Young (25 weeks), Ideal (44 weeks) and Old (68 weeks). Eggs from each maternal age, treatment and flock were collected and stored at 4 °C until incubation.

Birds from all treatments were fed identical, standard commercial crumbled pellet diets appropriate for rearing and lay and followed the same vaccination program (see supplementary material for further information). During rearing, room temperature was slowly decreased from 35 °C at day 1 to 21 °C by week 5, while lighting was decreased from 16L:8D at 20 lx to 8L:16D (lights on at 6:00 h) at 10 lx by 9 weeks. During the laying period, ambient temperatures were maintained at 21 °C and lighting schedule was 14L:10D at 20 lx for hens in all treatments. The males used for insemination were all housed individually in standard cages, followed the same lighting and temperature protocol as laying hens and were fed standard crumbled pellets.

#### Offspring management and data collection

Offspring were hatched at the University of Guelph’s Arkell Poultry Research Station using commercial grade incubators and hatchers (Nature Form, Jacksonville, FL). Chicks were sexed and individually wing-banded at hatch. Each cohort had four replicate groups of progeny (8 groups per treatment x maternal age combination; 120 groups in total, 7 males and 7 females/pen; N = 1680). Replicate groups of chicks were identically reared to 15 weeks in 20 in pens with wood shavings on the floor (3.72 m^2^) that had a perch (length: 155 cm) and were bedded with litter. The test order for the procedures described below were balanced across treatment and time of day in order to minimize any effects of circadian rhythm on the results.

#### Manual restraint test

At 9 weeks of age, 2 males and 2 females from each treatment replicate (N = 480) were subjected to a manual restraint test^30^, which aimed to measure a bird’s behavioural and physiological response to stress through the assessment of number of escape attempts and plasma corticosterone (CORT) concentration. All birds were individually tested in a quiet space adjacent to their home pens. For this, each tested bird was removed from its pen and placed on a table. The test started when the bird was put on its side while the test person simultaneously restrained its chest and legs using both hands. During a period of 5 min, each successful attempt to escape (i.e., struggle) was recorded. Whenever that happened, the bird was immediately repositioned, and the test continued. Immediately following restraint, a blood sample (3 ml) was drawn from the wing vein for CORT analysis before the bird was returned to the pen. For details on plasma extraction and enzyme immunoassay, see Supplementary Material.

#### Comb pecking wounds

Combs were assessed for pecking wounds according to the Welfare Quality protocol^31^, using a score of 0 to 2. In this score, 0 meant no evidence of pecking wounds, 1 meant less than 3 pecking wounds, and 2 meant 3 or more wounds. All birds (N = 1680) were scored at 9, 11 and 13 weeks of age by two trained observers blind to treatment.

#### Social feather pecking test

Social feather pecking tests have been traditionally used to measure predisposition for feather pecking in laying hens^32–34^. In our study, 4 groups of 5 unfamiliar pullets from different replicate pens of the same treatment were formed and placed in a novel and bright environment for 60 min (8 groups per treatment and maternal age; N = 600). Since we only had 4 replicates per treatment, each test group had 2 pullets from the same home pen and 1 pullet from each of 3 different pens. However, this was balanced across groups.

One day before testing at 14 woa, birds were spray painted on their backs with different colours in order to allow for individual identification on video analyses. On test day, the pullets were crated, moved to the test room and placed into an arena made of solid white panels with two doors located on opposite walls and black rubber mats on the floor. The arena was internally divided with two white panels, thus forming 4 equally sized areas (50 cm long × 50 cm wide × 200 cm high) that were simultaneously used during the test. This layout prevented groups from seeing each other but was not acoustically isolated. On top of each section of the arena, a bright LED lamp and a camcorder (Panasonic HC-V180K) were attached to the ceiling. The cameras faced the centre of the arena and provided a full aerial view of the space. Pullets were recorded for 60 min and were immediately returned to their home pen after testing.

Measurements of behaviour were analysed from the video recordings by two observers blind to treatment using Pocket Observer software (Observer XT, Noldus Information Technology, Wageningen, The Netherlands). Behaviour measurements are described in Table 1.

**Table 1:** Social feather pecking test. Ethogram used for behaviour observations.

Behaviour	Description
Aggressive peck	A determined downward pecking, usually at the head and neck followed by full body movement of the recipient. Each peck is an event
Feather pulling	Removal of a feather from another bird. Each pull is an event
Gentle peck	Gentle, individual nibbling action, usually at the tail/wing. Each peck is an event
Preening	The stroking of feathers with beak. A self-action. Recorded as total duration
Severe peck	A forceful peck usually directed at the back of a recipient not always resulting in an aversive reaction from recipient. Each peck is an event

### Experiment 2 (E2)

#### Parent stock

In our second experiment, we aimed to further investigate the effects of maternal age in layer breeders obtained from commercial settings. For this, fertile eggs were acquired from 9, unique, commercial Lohmann Selected Leghorn-Lite layer breeder flocks located in Ontario, Quebec and New Brunswick, Canada and from Pennsylvania, USA. Breeder flocks were loose-reared and housed in floor barns. At the time of egg collection, 3 of the flocks were Young (25–27 woa), 3 were Ideal (42–46 woa) and 3 were Old (68–72 woa). All of the fertile eggs were shipped to University of Guelph’s Arkell Poultry Research Station within the same week prior to egg setting, where subsequent egg storage, incubation and hatch occurred together, following the procedures previously described. From each parent flock, 2 replicate groups of progeny (6 groups per maternal age, 7 males and 7 females per group; N = 252) were identically reared to 41 days of age. Offspring were subjected to identical husbandry and housing as E1.

#### Manual restraint test

At 4 weeks of age, all birds (N = 252) were subjected to a manual restraint test. This time, the birds were physically restrained inside a cloth bag for 5 min prior to blood sample collection, following the methodology proposed by Wingfield^35^. Only measures of plasma CORT concentration were collected. All the birds were individually tested and immediately returned to their home pen.

### Data analyses

The Glimmix procedure of SAS 9.4 (SAS Institute, Cary, NC) was used to perform statistical analyses. The experimental design was an incomplete factorial, due to constraints of moving conventionally reared pullets into aviary housing as adults. Therefore, standard techniques for testing main effects of rearing, housing and their interaction were not possible. The basic statistical model for E1 included fixed effects of sex (when applicable), maternal treatment (interaction of rearing and housing), maternal age and maternal treatment by age. Random effects included cohort, home-pen nested within room and person applying the test when applicable. Contrast comparisons tested the main effects of maternal rearing (Av or CC) and housing (Av, CC or CF). Data from the manual restraint and social feather pecking tests were subjected to a log transformation. For comb pecking score, a multinomial distribution was used to test the effects of maternal age, treatment and sex, and contrasts and estimates were used to compare differences in the levels of fixed effects. Inter-observer reliability was calculated using Kendall’s Tau-b coefficient, where a score of 1.0 is considered a perfect relationship, and a score of 0.7 is considered acceptable^36^. In E2, we used a generalized mixed model to test the effects of sex and maternal age on CORT concentration. Random effects included flock of origin and home-pen of the tested bird nested within room. Tests for normality included Shapiro–Wilk and Anderson Darling measurements in conjunction with visual plots for all analyses and statistical significance was defined as p < 0.05 for both studies.

## Results

### Manual restraint

**Figure 2:**
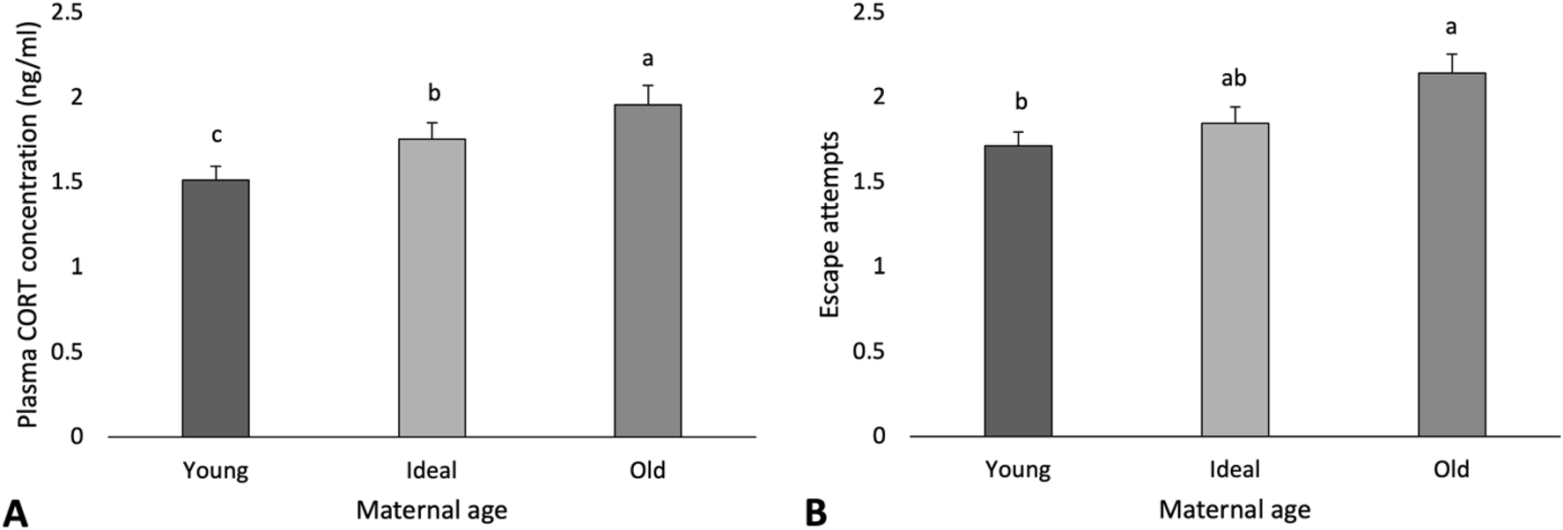
Manual restraint test. (**A**) Average plasma corticosterone concentration (ng/ml) and (**B**) average number of escape attempts displayed by maternal age. Statistical differences within tests are indicated by letters (a–c) (P < 0.05).

**Figure 3:**
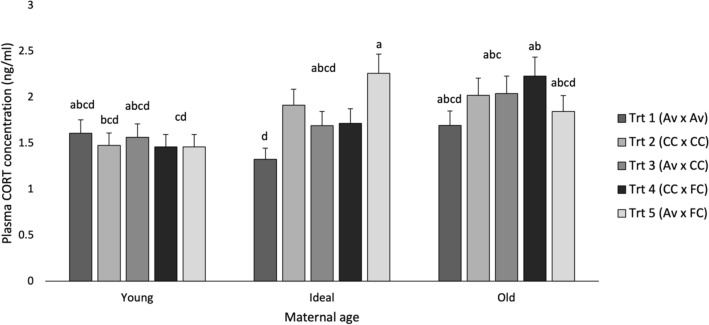
Manual restraint test. Effects of treatment and maternal age on plasma corticosterone concentration (ng/ml) displayed by maternal age. Statistical differences in plasma corticosterone concentration across treatments are indicated by letters (a–d) (P < 0.05).

Table 2 summarizes the P-values for manual restraint for E1 and E2. In E1, plasma corticosterone (CORT) concentration (P < 0.001) and number of escape attempts (P = 0.035) increased as mothers aged (Fig. 2). A treatment by maternal age interaction (P = 0.009; Fig. 3) showed that within the Ideal age, the offspring of mothers reared in aviaries and housed in furnished cages (Trt 5) produced more CORT than those both reared and housed in aviaries (Trt 1). Within Trt 5, the offspring of Young hens produced less CORT than the offspring of Ideal.

**Table 2:** Manual restraint test.

Manual restraint	E1		E2
Effect	Plasma corticosterone	Escape attempts	Plasma corticosterone
Maternal age	**< 0.001**	**0.035**	0.882
Treatment	0.085	0.589	–
Sex	0.887	**< 0.001**	**< 0.001**
Treatment × maternal age	**0.009**	0.764	–
**Contrasts**			
Rearing housing			
Av vs CC	0.297	0.814	–
Laying housing			
Av vs CC	**0.015**	0.899	–
Av vs FC	**0.007**	0.509	–
CC vs FC	0.740	0.342	–

Effects of maternal age, treatment and treatment by maternal age on plasma corticosterone concentration and escape attempts, followed by contrast comparisons of maternal rearing and laying environments. P-values less than 0.05 are bolded.

Still in E1, no effects of sex were observed on plasma CORT (P = 0.887), but females struggled more (2.11 ± 0.2 times/5 min) than males (1.69 ± 0.2 times/5 min) during the test. Further pre-planned comparisons showed that the offspring of mothers housed in aviaries (1.53 ± 0.1 ng/ml) during laying produced less CORT than the offspring of hens housed in either conventional (1.78 ± 0.2 ng/ml) or furnished cages (1.82 ± 0.2 ng/ml). No effects of treatment were found for escape attempts (P = 0.589).

E2 offspring showed no effects of maternal age on plasma CORT concentration (P = 0.882). However, a sex effect was found (P < 0.001), with males (2.56 ± 0.1 ng/ml) producing more CORT than females (1.68 ± 0.06 ng/ml) in reponse to restraint.

### Comb score

Comb score was affected by maternal age when offspring were 11 weeks of age (P = 0.001). Offspring from Young mothers had fewer non-injured combs (39%) than those from Ideal (56%) and Old (51%) mothers (Supplementary Table S1). No effects of treatment were observed (Table 3), and males consistently displayed more injured combs than females.

**Table 3:** Comb score.

Comb score	P-values		
Effect	9 weeks	11 weeks	13 weeks
Maternal Age	0.152	**0.001**	0.690
Treatments	0.552	0.557	0.557
Sex	**< 0.001**	**< 0.001**	** 0.001**
**Contrasts**			
Rearing housing			
Av vs CC	0.567	0.925	0.890
Laying housing			
Av vs CC	0.679	0.597	0.678
Av vs FC	0.235	0.420	0.153
CC vs FC	0.153	0.092	0.210

Effects of sex, treatment, maternal age and treatment by maternal age on comb score, and contrast comparisons by rearing and laying environments. Data are summarized in the text and presented in Supplementary Table S1). P-values less than 0.05 are bolded.

### Social feather pecking test

**Figure 4:**
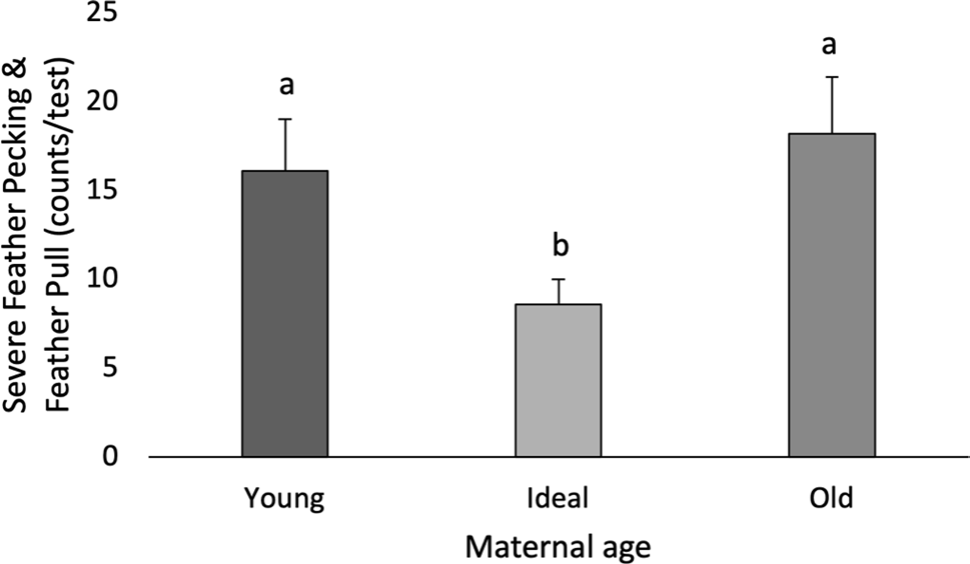
Social feather pecking test. Number of severe feather pecking and feather pulling displayed by maternal age.

Since not many hens performed either severe feather pecking or feather pulling, we combined both measurements into one category (Severe Feather Peck & Feather Pulling). Maternal age affected the occurrence of severe pecking and feather pulling (P = 0.016); offspring of Ideal mothers performed less pecking compared to offspring from Young and Old (Fig. 4). Maternal treatment, however, did not affect any of the observed behaviours in the offspring (Table 4). Contrast comparisons showed that the offspring from hens reared in CC performed more preening (30.15 ± 6.8 s) behaviour than Av (19.67 ± 4.67 s).

**Table 4:** Social feather pecking test.

Induced feather pecking	P-values			
Effect	Preening	Aggressive peck	Gentle peck	Severe peck & feather pulling
Maternal age	0.458	0.775	0.172	**0.016**
Treatment	0.145	0.662	0.863	0.899
Treatment × maternal age	0.777	0.705	0.070	0.234
**Contrasts**				
Rearing housing				
Av vs CC	**0.016**	0.438	0.309	0.466
Laying housing				
Av vs CC	0.785	0.345	0.697	0.642
Av vs FC	0.373	0.783	0.538	0.885
CC vs FC	0.434	0.425	0.797	0.697

Effects of maternal age, treatment and treatment by maternal age on preening, aggressive peck, gentle peck and severe peck and feather pulling, followed by contrast comparisons of rearing and laying environments. P-values less than 0.05 are bolded.

## Discussion

Maternal experience effects have been mostly studied in wild birds^2^, where the outcomes (mainly due to predation and food availability) are suggested to be adaptive if mother and offspring share similar environments; or detrimental if their environments differ (“environmental matching hypothesis”)^37–39^. In passerine birds, for example, maternal stress was found to decrease hatching weight^40–42^, begging behaviour^40,42^ and late embryonic vocalization^40^. Similarly, the offspring of yellow-legged gulls exposed to a predator decoy were not only lighter than control chicks, but also crouched faster after hearing a playback with alarm calls^43^. Producing smaller and more fearful (i.e., alert) offspring might be positive in nature, when food availability is low or predation risk is high. However, an increase in fearfulness or stress reactivity in birds kept in artificial environments is generally detrimental, regardless of their housing environment. Although maternal effects in response to acute stressors have been demonstrated in domesticated laying hens^11,44^ to our knowledge, this is the first study to explore the effects of maternal experience of different housing system during early life (rearing) and during the laying period.

Maternal age effects have been reported for a variety of response variables across several species. In invertebrates and mammals, they are frequently associated with increased mortality risk and infertility of offspring^45^. However, in avian species, inconsistency across results seems to be more frequent^24,26,45^, possibly due to natural changes in egg composition according to hen’s age^22^ and environmental context^4^. Research in broiler breeders has linked maternal age to changes in yolk fatty acid profile^46,47^ and late embryonic mortality, hatchability, and body weight^48^. In addition, a previous report from our research group on the same birds used in E1 showed advanced maternal age was associated with less egg yolk testosterone and increased fearfulness in the offspring^23^, allowing for a potential relationship between the results reported here and maternal age effects due to changes in yolk testosterone concentration.

In E1, we found that older mothers had more reactive offspring during the restraint test. As mothers aged, their offspring had progressively higher concentrations of plasma CORT and struggled more. However, the maternal effect on plasma CORT following restraint was not replicated in E2, possibly due to methodological differences, including the type of restraint and age at the time of testing. Since the hypothalamic–pituitary–adrenal (HPA)-axis is a set of components that matures over time, results are naturally expected to be age dependant^49,50^. Additionally, blood was collected within a relatively short time period, only five minutes after the start of restraint, and values were low compared to those reported in other studies^28^. CORT levels following restraint increase over time, with samples collected at 10–15 min following the start of restraint considered to reflect maximum response values in laying hens^30^. Another point to be considered is the smaller experimental size in E2 compared to E1. Unfortunately, our study was performed during an Avian Influenza outbreak across North America that severely limited access to eggs from commercial breeder flocks. The main goal of running two experiments was to access E1’s replicability in commercial-like genetics. In commercial parent stock, female breeders are derived from different genetic lines than male breeders, and the commercial laying hen is the combination of both of their genetics. While we used commercial laying hens as a model for layer breeders in E1, we directly assessed the progeny of layer breeders in E2. Therefore, differences between experiments could be genetically driven, suggesting that layer breeders may be more resilient to maternal effects, as reported in previous studies^11,29^. Additionally, the rearing, housing, diet, management and social environments of breeder flocks (usually comprising thousands of birds and allowing a natural mating system of hens and roosters^12^) can also affect the endocrine status of the hen^51^, thereby affecting the hormone content of the layer breeder’s eggs^52^. Maternal nutrition can also impact the composition of the egg, and commercial breeder flocks are usually fed diets with different nutrient levels over the course of their laying period^12^ (phase feeding). This was not done in our experimental flock in E1. All of these factors would likely add variation, potentially masking maternal age effects.

Still in E1, we found effects of maternal age on injurious behaviour, including a higher occurrence of feather pulling and severe pecking in offspring of Young and Old mothers during the social feather pecking test; and worst comb condition at 11 woa in offspring from Young mothers. These tests reflect different aspects of social behaviour. The social feather pecking test was developed to identify individuals that are prone to feather pecking^32–34^, a multifactorial aberrant behaviour with many contributing factors, including genetics, early-life experience and various neurobiological pathways^53^. Differently, comb score reflects the occurrence of aggressive behaviour occurring in the birds’ home environment. Although undesirable, aggressive pecking is a normal aspect of social development and functions to obtain resources, defend territory or establish social hierarchy^54^. Interestingly, the reported age for the establishment of social hierarchies in laying hens is around 9 to 12 woa^55^, coinciding with the maternal age effect observed at 11 woa in offspring from Young mothers. Thus, our results suggest that maternal age affects different aspects of social behaviour in laying hens, including propensity to feather pecking and aggression during establishment of a social hierarchy.

A maternal age by housing treatment interaction was only observed for one measure (plasma corticosterone), at one maternal age (Ideal), suggesting that the effects of early and current maternal housing experience might have a greater effect, or only are expressed, at certain maternal ages. Contrast comparisons showed that the offspring of mothers reared in Av displayed less preening behaviour during the social feather pecking test than CC; while the offspring of mothers housed in Av as adults had offspring that produced less corticosterone in response to manual restraint than CC and FC. Preening behaviour is displayed in several contexts: as grooming and comfort behaviour for maintenance of feather condition or as a displacement activity, related to situations of conflict, frustration and stress^56^. Therefore, results suggest that the offspring of Av-reared mothers were less emotional under a stressful situation, and the offspring of Av-housed mothers were physiologically less stressed by manual restraint.

Although there has been an increased scientific interest in the effects of maternal effects over the past decade, many questions remain unresolved, such as physiological pathways, susceptible periods, duration and consistency of effects^44^. Moreover, discrepancies across studies are often observed, as maternal effects are highly context dependent. Research suggests that rearing environment of laying hens can cause maternal effects through changes in DNA methylation profiles^57^; and stressors experienced during the laying period may be related to *in ovo* hormone-mediated maternal effects^58^. In view of this, we acknowledge the highly complex nature of this subject and encourage further research on both fundamental and applied levels.

Lastly, we did not observe any sex interaction with maternal age or maternal housing treatment combinations. This seemed rather surprising, as maternal effects are often sex-dependent^44^. However, we found sex differences in behavioural response to restraint^59,60^, comb score, which is an indirect measure of aggression^61,62^ and stress response^63,64^. Sexually dimorphic behaviour is mainly related to the effects of gonadal hormones, androgens and estrogens, on the nervous system^65^. Individually and combined, these hormones can organize the neuronal circuitry involved in behavioural functions, including the serotonergic system^66,67^, responsible for traits of fear^68^, anxiety^69^, stress response^70^ and aggression^71^.

In conclusion, our study indicates that maternal age and maternal environment can affect offsprings' behaviour and physiology when commercial hens are used as model for breeding chickens. Maternal age was shown to increase both the endocrine and behavioural response of the offspring to manual restraint, and to affect different aspects of injurious social behaviour such as comb wounds, which may be linked to the establishment of a social hierarchy, and susceptibility to feather pecking. Both maternal rearing and housing systems affected the offspring, but in different ways and possibly through different mechanisms. The offspring of mothers reared in Av were less emotional as indicated by less displacement preening in the social feather pecking test, whereas the chicks of mothers housed in aviaries at the time of fertilization had a lower stress response.

In light of our findings, we highlight the importance of maternal age and maternal environment on the behaviour and physiology of laying hens. In addition, we strongly recommend researchers to start reporting the age and housing condition of their birds’ parent stock even if this is not their main focus of research. By doing this, they can provide data for future studies (e.g., meta-analysis) that will further clarify the extent of maternal effects. Likewise, we encourage farmers to note their flocks' parents' age to identify possible behaviour patterns and eventually anticipate productive issues.

## Supplementary Information

Supplementary Information.

## Data Availability

The data that support the findings of this study are available from the corresponding author upon reasonable request.
