# Supplementary material for: Maternal age and maternal environment affect stress reactivity and measures of social behaviour in laying hens
Source: Sci Rep. 2021 Sep 1;11:17499. doi: 10.1038/s41598-021-96323-6 (PMC8410862; doi:10.1038/s41598-021-96323-6)
Supplement: Supplementary file 1 — Supplementary Information. [file 41598_2021_96323_MOESM1_ESM.docx]

**Supplementary material**

***Housing systems:***

*Conventional brooding cages:* Ford Dickinson, Mitchel, Ontario, Canada; 16 pullets/cage during 0-6 weeks with a space allowance of 145 cm2/pullet followed by 8 pullets/cage during 6-16 weeks with a space allowance of 290 cm2/ pullet; total cage area = 2,322 cm^2^. The cages had wire floors and sides with nipple waterer and feed trough.

*Pullet aviary rearing system:* Farmer Automatic Portal, Clark Ag Systems, Caledonia, Ontario, Canada; 756 pullets/aviary enclosure; system space allowance of 285 cm^2^/pullet during 0-6 weeks; total system + outer platforms + litter space allowance of 754 cm^2^/pullet during wks 6-16. The aviary provided access to 183,272 cm^2^ of floor area, multiple perches at different heights, and a suspended platform (32,371 cm^2^) that was gradually raised vertically as the pullets grew older (total system area: 215,643 cm^2^). At 6 weeks of age, access to the litter area covered in wood shavings (235,767 cm^2^) and 9 elevated terraces with wire floors (118,887 cm^2^) was provided, increasing the total system area to 570,297 cm^2^. The main section of the aviary had wire floors, rows of nipple drinkers and feed troughs.

*Standard conventional cages:* 12 equal sized wire-floored cages (8 hens/cage; total area = 4,025 cm^2^, 503 cm^2^/hen) containing one feeder and two nipple drinkers. All cages were located in the same room.

*Furnished cages:* 12 Farmer Automatic Enrichable (Furnished) Cages (Clark Ag System) of equal size (60 hens/cage, total area = 41,296 cm^2^, 688 cm^2^/hen). The furnished cages were located in two similar rooms and provided a curtained nest area (94 cm^2^/hen), 10 cm high perches (15 cm^2^/hen), and a smooth plastic scratch area (42 cm^2^/hen). The furnished cages had wire floors, nipple drinkers and a feed trough.

*Aviary systems:* 2 Farmer Automatic Loggia Aviaries (Clark Ag System) located in 2 equal sized rooms (270 hens/room). Each aviary (122,675 cm^2^) had two tiers with wire floors, nests, a row of nipple drinkers and a feed trough and had an accompanying litter area on the floor that was covered with wood shavings (240,443 cm^2^; total area = 363,118cm^2^, 1,344 cm^2^/hen).

***Feed and vaccination programs***

Birds were fed commercial fine crumble starter until 6 weeks of age (2,900 kcal AME, 21.0% CP, 1.06% Ca and 0.77% P) then coarse crumbles (2900 kcal ME, 18.0% CP, 1.00 % Ca and 0.78 % P). The vaccination program provided at the hatchery included bronchitis (spray), Marek’s disease (injection) and Immucox (gel droplet). In addition, birds were vaccinated for Newcastle-bronchitis vaccine (spray) at 3 weeks, ILT Vectormune FP-LT-AE (wing web) at 6 weeks and Newcastle-bronchitis at 10 (spray) and 16 (intramuscular) weeks.

***Corticosterone analysis: Plasma Extraction***

Steroid hormones were extracted from plasma with 100% EtOH. In brief, EtOH was added to each plasma sample at a 5:1 ratio. Samples were vortexed for 3 minutes and centrifuged (5 min, 20C, 800G). Samples were then flash frozen at -80C and the EtOH supernatant containing steroid hormones decanted and dried under air in a heated water bath. Extracts were reconstituted in ½ volume of original volume of plasma extracted of trizma buffer for assay (see below).

***Corticosterone analysis: Enzyme Immunoassay***

Plasma corticosterone was quantified using an enzyme immunoassay. For this, microtitre plates (Fisher Scientific Canada, Ottawa, ON) were coated with affinity purified goat anti-rabbit gamma globulin (25 μg/plate; Sigma Chemicals, St. Louis, MI) dissolved in coating buffer (0.015M Na2CO3, 0.035M NaHCO3; pH 9.6) and incubated overnight at room temperature. Wells were emptied and refilled with trizma buffer (0.02M Trizma, 0.300M NaCl, 0.1% BSA; pH 7.5) and stored at room temperature for at least 1 hour prior to use to block non-specific binding. Coated plates were washed (0.04% Tween 20) and sample and standards were dispensed. Horseradish peroxidase-labeled corticosterone (supplied by CJ Munro; UC Davis) was dispensed followed by anti-corticosterone antibody (CJM006; supplied by CJ Munro). Following overnight incubation at room temperature, plates were washed and substrate solution (0.5 ml of 0.016M tetramethylbenzidine in dimethylsulphoxide and 100 μl of 0.175M H2O2 diluted in 24 ml of 0.01M C2H3O2Na; pH 5.0) was added to each well. After incubation (45 min, room temperature) the enzyme reaction was stopped with 3M H2SO4 and the optical density was measured at 450 nm (reference 595 nm). The standard curve of CORT ranged from 3.9 – 500 pg/well. Inter and intra-assay coefficients of variation were <15%. Serial dilutions of pooled plasma extract yielded a dilution curve that was parallel to the standard curve.

**Supplementary Table S1. Comb score.** Number of birds per weeks of age and score, displayed by maternal age, sex and treatment.

| **Comb Score**  **Effects** |  | **9 weeks** | | | | **11 weeks** | | | | **13 weeks** | | | |
| --- | --- | --- | --- | --- | --- | --- | --- | --- | --- | --- | --- | --- | --- |
|  |  | **N** | **Score** | | | **N** | **Score** | | | **N** | **Score** | | |
|  |  |  | **0** | **1** | **2** |  | **0** | **1** | **2** |  | **0** | **1** | **2** |
| **Mat. Age** | Young | 400 | 189 | 130 | 81 | 400 | 157^a^ | 165 | 78 | 399 | 160 | 162 | 77 |
|  | Ideal | 398 | 195 | 110 | 93 | 398 | 222^b^ | 101 | 75 | 397 | 150 | 163 | 84 |
|  | Old | 397 | 224 | 86 | 87 | 398 | 202^b^ | 111 | 85 | 398 | 179 | 112 | 107 |
| **Sex** | Female | 594 | 513^a^ | 79^a^ | 2^a^ | 594 | 463^a^ | 123^a^ | 8^a^ | 592 | 356^a^ | 197^a^ | 39^a^ |
|  | Male | 602 | 95^b^ | 247^b^ | 260^b^ | 602 | 118^b^ | 254^b^ | 230^b^ | 602 | 133^b^ | 240^b^ | 229^b^ |
| **Treatment** | 1 (AvxAv) | 238 | 122 | 73 | 43 | 238 | 116 | 71 | 51 | 237 | 104 | 83 | 50 |
|  | 2 (CCxCC) | 240 | 124 | 67 | 49 | 240 | 119 | 80 | 41 | 240 | 104 | 87 | 49 |
|  | 3 (AvxCC) | 240 | 127 | 62 | 51 | 240 | 124 | 75 | 41 | 239 | 99 | 84 | 56 |
|  | 4 (CCxFC) | 240 | 120 | 62 | 58 | 240 | 111 | 80 | 49 | 240 | 92 | 93 | 55 |
|  | 5 (AvxFC) | 238 | 115 | 62 | 61 | 238 | 111 | 71 | 56 | 238 | 90 | 90 | 58 |

Results are presented by effect (maternal age, sex, treatment), offspring age (9, 11, 13 weeks) and comb score (0,1,2). Counts with different letter superscripts (a, b) within the same effect and score differ (P < 0.05).
